# Non-Lethal Concentration of MeHg Causes Marked Responses in the DNA Repair, Integrity, and Replication Pathways in the Exposed Human Salivary Gland Cell Line

**DOI:** 10.3389/fphar.2021.698671

**Published:** 2021-08-25

**Authors:** Lygia Sega Nogueira, Carolina P. Vasconcelos, Jessica Rodrigues Plaça, Geovanni Pereira Mitre, Leonardo Oliveira Bittencourt, Maria Sueli da Silva Kataoka, Edivaldo H. C. de Oliveira, Rafael Rodrigues Lima

**Affiliations:** ^1^ Laboratory of Functional and Structural Biology, Federal University of Pará, Belém, Brazil; ^2^ Laboratory of Cell Culture and Cytogenetics, Environment Section, Evandro Chagas Institute, Ananindeua, Brazil; ^3^ Regional Blood Center at University Hospital of the Ribeirão Preto Medical School of University of São Paulo, Ribeirão Preto, Brazil; ^4^ School of Dentistry, Federal University of Pará, Belém, Brazil

**Keywords:** toxicology, salivary gland, transcriptome, methylmercury, biomarker

## Abstract

In Brazilian northern Amazon, communities are potentially exposed and vulnerable to methylmercury (MeHg) toxicity through the vast ingestion of fish. *In vivo* and *in vitro* studies demonstrated that the salivary glands as a susceptible organ to this potent environmental pollutant, reporting alterations on physiological, biochemical, and proteomic parameters. However, the alterations caused by MeHg on the gene expression of the exposed human salivary gland cells are still unknown. Therefore, the goal was to perform the transcriptome profile of the human salivary gland cell line after exposure to MeHg, using the microarray technique and posterior bioinformatics analysis. The cell exposure was performed using 2.5 µM MeHg. A previously published study demonstrated that this concentration belongs to a range of concentrations that caused biochemical and metabolic alterations in this linage. As a result, the MeHg exposure did not cause lethality in the human salivary gland cells line but was able to alter the expression of 155 genes. Downregulated genes (15) are entirety relating to the cell metabolism impairment, and according to KEGG analysis, they belong to the glycosphingolipid (GSL) biosynthesis pathway. On the other hand, most of the 140 upregulated genes were related to cell-cycle progression, DNA repair, and replication pathway, or cellular defenses through the GSH basal metabolism. These genomic changes revealed the effort to the cell to maintain physiological and genomic stability to avoid cell death, being in accordance with the nonlethality in the toxicity test. Last, the results support in-depth studies on nonlethal MeHg concentrations for biomarkers identification that interpret transcriptomics data in toxicological tests serving as an early alert of physiological changes *in vitro* biological models.

## Introduction

Methylmercury (MeHg) is an organic form of mercury considered a potent environmental pollutant (Clarkson, 2002). In Brazilian northern Amazon, the artisanal small-scale gold mining (ASGM) is the main source of anthropogenic mercury emissions and contamination (Hacon et al., 2020). Communities whose diet involves vast ingestion of fish are potentially exposed and vulnerable to MeHg toxicity, which endangers the food security and livelihoods of traditional communities (Crespo-López et al., 2009; Crespo-Lopez et al., 2016; Hacon et al., 2020). Contaminated seafood is widely absorbed in the gastrointestinal tract and ubiquitously distributed to the body with an extensive effect in several organ systems (Kershaw et al., 1980). The central nervous system (CNS) is considered the principal target of MeHg due to the high affinity of this metal to the brain (Antunes Dos Santos et al., 2016); however, *in vivo* and *in vitro* studies have demonstrated that the salivary glands and cells from the oral cavity are also susceptible to MeHg toxicity (Bittencourt et al., 2017; Farias-Junior et al., 2017; Nogueira et al., 2019; Nascimento et al., 2020; Nogueira et al., 2021).

The salivary glands are essential for oral cavity health, playing the direct impact on the salivary production and also an important role as a diagnostic window into human disease (Saitou et al., 2020). The sub-chronic (35 days) and chronic (60 days) exposure of rats to MeHg-generated oxidative imbalance in the parotid and submandibular glands, by the depression of total antioxidant capacity and increased lipid peroxidation (Bittencourt et al., 2017; Farias-Junior et al., 2017). Besides that, the MeHg was able to induce changes in the proteomic profile with impairments on structural components of the cytoskeleton, metabolic pathways, and oxidative parameters (Bittencourt et al., 2017).

Through *in vitro* experiments, the human salivary gland cell line exposed to MeHg had compromised their metabolism and oxidative balance (Nogueira et al., 2021). A low dose of MeHg (0.25 µM) induced an increase in metabolism. A low dose of MeHg (0.25 µM) induced an increase in metabolism. This concentration represents the LC50 previously calculated in the toxicity tests performed with the human salivary gland cell line, and also represents the Hg accumulated in the parotid and submandibular glands of rats exposed chronically *in vivo* (Bittencourt et al., 2017; Nogueira et al., 2021). However, the *in vitro* exposure to a concentration 10-fold higher (2.5 mM) caused depression of cell metabolic status, generating an oxidative stress status by the depression of the GSH:GSSG rate and a significant increase of lipid peroxidation and protein carbonyl (Nogueira et al., 2021). Interestingly, although it is known that one of the exposure to MeHg causes DNA strand breaks (Crespo-López et al., 2007; Crespo-López et al., 2009; Antunes Dos Santos et al., 2018), this effect has not been observed in the exposed human salivary gland cell line to 2.5 µM MeHg.

To deepen the understanding of the mechanisms related to exposure of human salivary gland cells line to MeHg, the present study aimed to investigate the transcriptome profile of these cells after exposure to a nonlethal concentration of MeHg. The methodology performed was the microarray, which allowed the description in a genome-wide level of alterations in 26.000 genes through a full range of RNA molecules expressed by the cells.

## Material and Methods

### Cell Culture

The human salivary glands cells line, obtained from nonneoplastic cells isolated from the submandibular salivary gland and cultured in a Dulbecco’s modified Eagle’s medium (DMEM) and Ham’s F-12 nutrient medium (1:1), supplemented with 10% fetal bovine serum (FBS), 100 U/ml penicillin, and 100 μg/ml streptomycin, incubated at 37°C in a 5% CO_2_ environment. The medium was changed every 48 h. When cells became fully confluent, they were detached from the flask using 0.25% trypsin solution, and seeded into new flasks.

### MeHg Exposure

**FIGURE 1:**
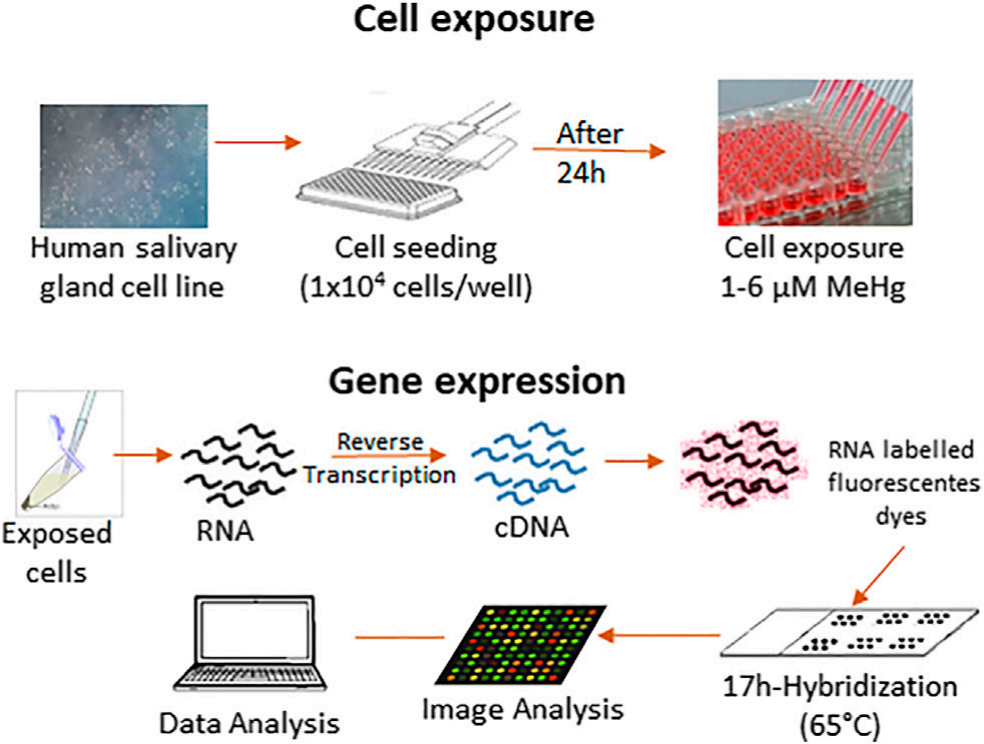
Schematic representation of the research methodology.

Cells were seeded in a concentration of 1 × 10^4^ cells/well in a 96-well/plate and cultured in a fresh culture medium for 24 h. After this, the medium was replaced by the exposure solution containing MeHg with no FBS supplementation, plus a control treatment (no metal addition). We used a concentration of 2.5 µM MeHg, which represents 10-fold higher the mercury accumulation found in the parotid and submandibular glands of rats chronically *in vivo* exposed to this compound (∼0.06 ppm; Bittencourt et al., 2017). At the end of the exposure time (24 h), the medium was removed, and the cells were washed with the EDTA solution (10 mM) to remove possible loosely bound mercury of the cell surface. Following that, cells were detached using trypsin and centrifuged (1000 g, 3 min) to proceed with the further analysis. Figure 1 represents a general research design and scheme of this study.

### Cell Viability

Percentage of viable cells from the total number of exposed cells was determined using the trypan Blue (0.04%) exclusion assay. The exclusion test is based on dye permeability in compromised cell membranes due the cell death. If cells have their metabolic integrity maintained, the dye is not permeable to their membrane. In this study, after 2.5 µM MeHg exposure, the exposed cells were re-suspended in a fresh culture medium, and 5 µL of this homogenate was added into 5 µL of trypan blue solution. Following that, the cells were counted under a light microscope using the Neubauer chamber (×200 magnifications). The results were expressed in percentage (%).

### Gene Expression

#### RNA Extraction

The human salivary gland cell lines were exposed as described above. At the end of 24 h, the cells were detached, and the pellet resulted from centrifugation had their mRNA extraction performed according to the SV total RNA isolation system kit assay from Promega^®^. Cells were homogenized in RNA lysis buffer contained beta-mercaptoethanol and transferred into a new tube containing RNA dilution buffer. After being centrifuged, samples were transferred into a clean tube, and 95% ethanol was added. Next step, samples were transferred into a spin column and three steps of centrifugation (1 min) occurred. First, using the RNA wash solution, next DNAse stop solution, and finally RNA wash solution. The RNA extracted was eluted into an elution tube using 15 µL of nuclease-free water. The RNA quantification was performed using TapeStation 4200 (Agilent Technologies), and the A_260/280_ ration was analyzed by a Nanodrop ND-1000 UV-VIS 3.2.1 version. The purified RNA was stored at −80°C to further gene expression assay.

#### Gene Expression Analysis–Microarray

The microarray analysis was performed according to the “One-color microarrays-based gene expression analysis” (Agilent technologies, EUA) protocol. This protocol was applied in nonexposed (control) and 2.5 µM MeHg exposed cells. Briefly, the obtained RNA was used as a template to synthesize the first cDNA, using a reverse transcription assisted by T7 RNA polymerase. A cRNA was transcripted from the second cDNA strand. Following that, the 3-cyanine was incorportated in the cRNA, and purified with the aid of the *RNeasy mini spin kit assay*. At the end, the cRNA was quantified by spectrophotometry, when the A_260/280_ ration and concentration were also analyzed (Nanodrop ND-1000 UV-VIS 3.2.1 version). For the hybridization, fragmentation mix was added to the RNA, and incubated at 60°C for 30 min 25 µL of 2x-RPM hybridization buffer were added to each sample, at 4°C. Finally, 40 µL of each sample was added to the hybridization slide, and left in the hybridization oven for 17 h, 10 rpm and 65°C. After that, the slides were washed and scanned immediately on the microarray Scanner (Agilent, G4900DA). Data were extracted from the raw microarray image file using the software Feature Extraction v10.10 and analyzed by GeneSpring GX (Version 11.0) software.

#### Bioinformatic Analyses

Differentially expressed genes were identified based on an absolute log2 fold change level >1 and the *p*-value adjusted by FDR <0.05. Quality control, quantile normalization, and batch effects removal were performed using the limma package. The over-representation analysis for differently expressed genes of gene ontology (GO) terms or KEGG pathways were also performed using the limma package. Over-represented *p*-values were adjusted by the Bonferroni method, and only adjusted *p*-values < 0.05 were considered.

## Results

### Cytotoxicity

The cell viability of the human salivary gland cell line exposed to 2.5 µM MeHg indicated a value of 87.5 ± 3.8%, while the nonexposed cells a value of 93.5 ± 0.4% (control). According to the statistical analysis, there are no differences between groups, showing a nonlethal MeHg concentration for the exposed salivary gland cell line at least for the 24 h tested.

### Modulation of Transcriptomic Profile by MeHg Exposure

**FIGURE 2:**
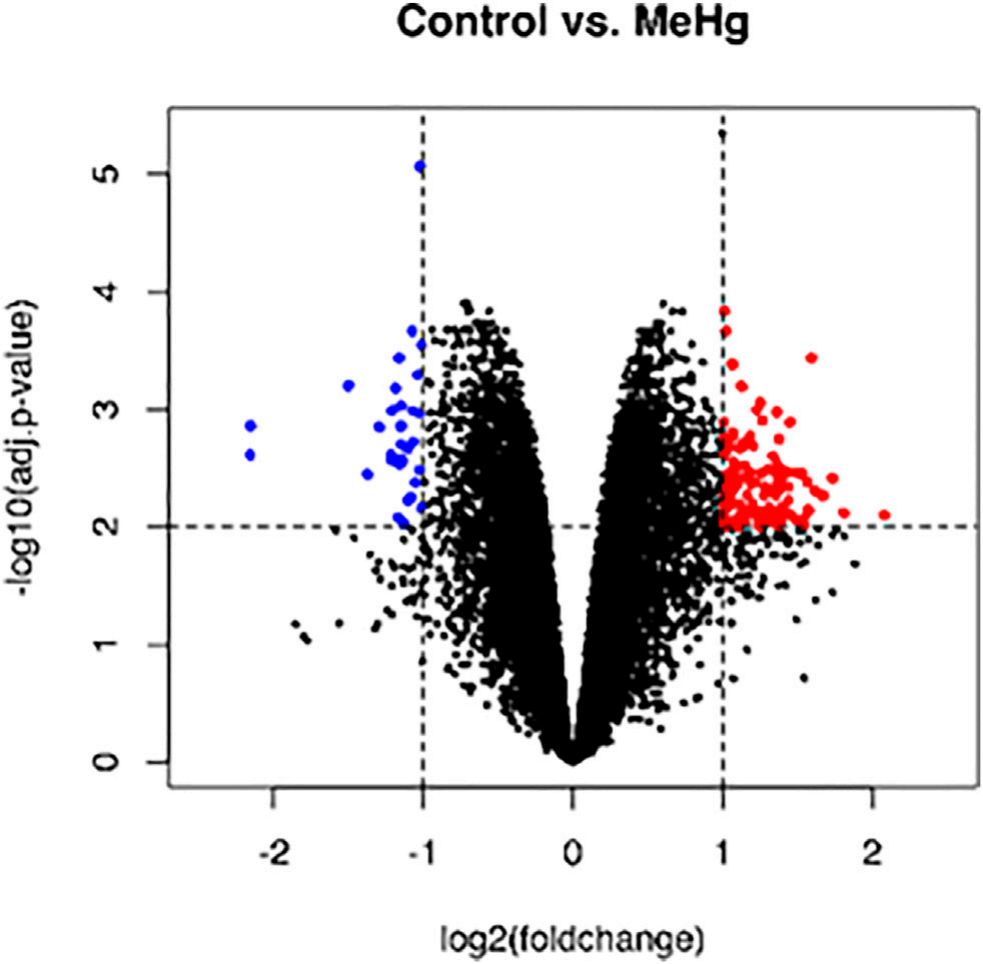
Global profile of gene expression of the exposed human salivary gland cells line to 2.5 µM of MeHg vs. nonexposed cells. The blue dots in the volcano plot represent downregulated genes, and red dots represent upregulated genes. Data were expressed as log2FC > 1 or < −1 and analyzed by *t*-Student test, adopting adj. *p* < 0.01 (*n* = 5/each group).

**FIGURE 3:**
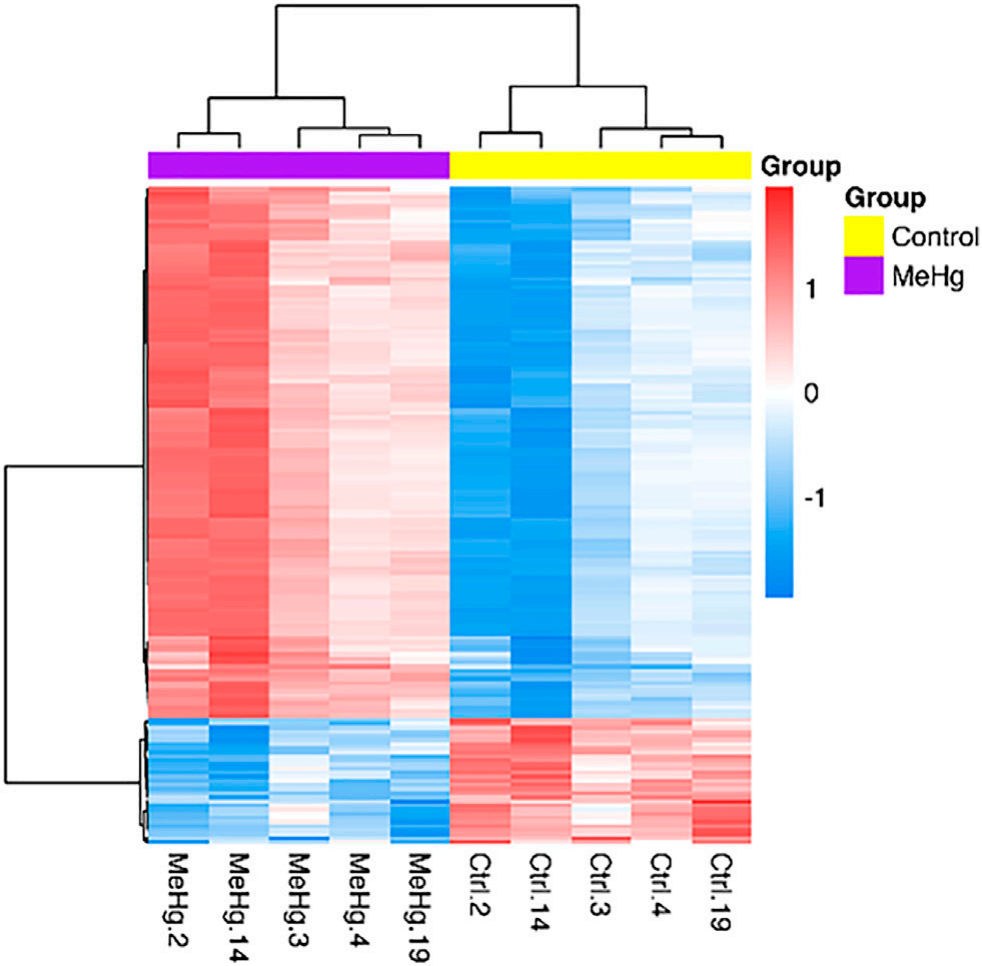
Differentially expressed genes in exposed human salivary gland cells line to 2.5 µM of MeHg vs. nonexposed cells (MeHg vs. Control) is demonstrated through the heat map. The dendrograms represent hierarchical cluster relationship between genes (left side) and among samples (upper), based on Pearson’s correlation; log2FC > 1 or < −1. log2fold change >1.5 or < −1.5; adj.; cutoff adj. *p*-value: < 0.01.

The whole transcriptome profile of the exposed human salivary gland cell line showed modulation of 155 genes, in which 140 were found upregulated and 15 downregulated, as observed in Figures 2 and 3 (log2fold change >1 or < −1; adj. *p*-value < 0.01). In Table 1, we highlighted the top 10 ranked genes with the highest (upregulated), and lowest (downregulated) fold changes. Figure 2 represents the distribution of differentially expressed genes in a volcano plot.

**TABLE 1:** Top 10 genes upregulated and top 10 downregulated in the exposed human salivary gland cells line to 2.5 µM of MeHg vs. nonexposed cells (MeHg vs. Control).

**Gene name**	**logF**C	**Adjusted *p* val**
**KRT34**	2.08	0.007
Keratin 34		
**CDC45**	1.81	0.007
Cell division control protein 45 Homolog		
**WDR62**	1.67	0.005
Microcephaly, primary autosomal recessive 2		
**POLQ**	1.62	0.004
DNA polymerase theta		
**CHAC2**	1.59	0.0003
Glutathione-specific gamma-glutamylcyclotransferase 2		
**FAM111B**	1.57	0.007
Cancer-associated nucleoprotein		
**ERCC6L**	1.56	0.004
DNA excision repair protein ERCC-6-like		
**SPC25**	1.54	0.008
Kinetochore protein Spc25		
**ZWINT**	1.53	0.009
ZW10-interacting kinetochore protein		
**NETO2**	1.53	0.003
Neuropilin and tolloid-like 2		
**C22orf43**	−1.17	0.002
Chromosome 22 open reading frame 43		
**PBXIP1**	−1.18	0.0006
Pre-B-Cell leukemia transcription factor-interacting protein 1		
**VSX1**	−1.20	0.001
Transcription factor VSX1		
**BHLHE23**	−1.21	0.002
Basic helix-loop-helix family member E23		
**KLHL24**	−1.21	0.002
Kelch like family member 24		
**C9orf62**	−1.29	0.001
Chromosome 9 open reading frame 62		
**ISLR**	−1.37	0.003
Immunoglobulin superfamily containing leucine rich repeat		
**LOC100507759**	−1.50	0.0006
**NKX2-5**	−2.15	0.001
NK2 Homeobox 5		
**C10orf10**	−2.15	0.002
DEPP1 autophagy regulator		

**FIGURE 4:**
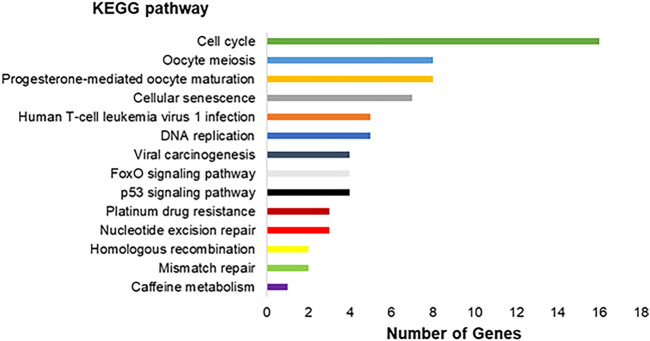
Molecular interaction, networks, and respective number of differentially expressed genes found in the transcriptomic analysis in the exposed human salivary gland cells line to 2.5 µM of MeHg vs. nonexposed cells (MeHg vs. Control) represented by KEGG pathways.

The genes modulated are involved in 14 different pathways, as observed in Figure 4. The genes are involved in pathways as *Cell cycle, Oocyte meiosis, Progesterone-mediated oocyte maturation, Cellular senescence, Human T-cell leukemia virus one infection*, and other nine pathways.

#### Analysis of Gene Ontology of the HSG Cells Transcriptome After the MeHg Exposure

**FIGURE 5:**
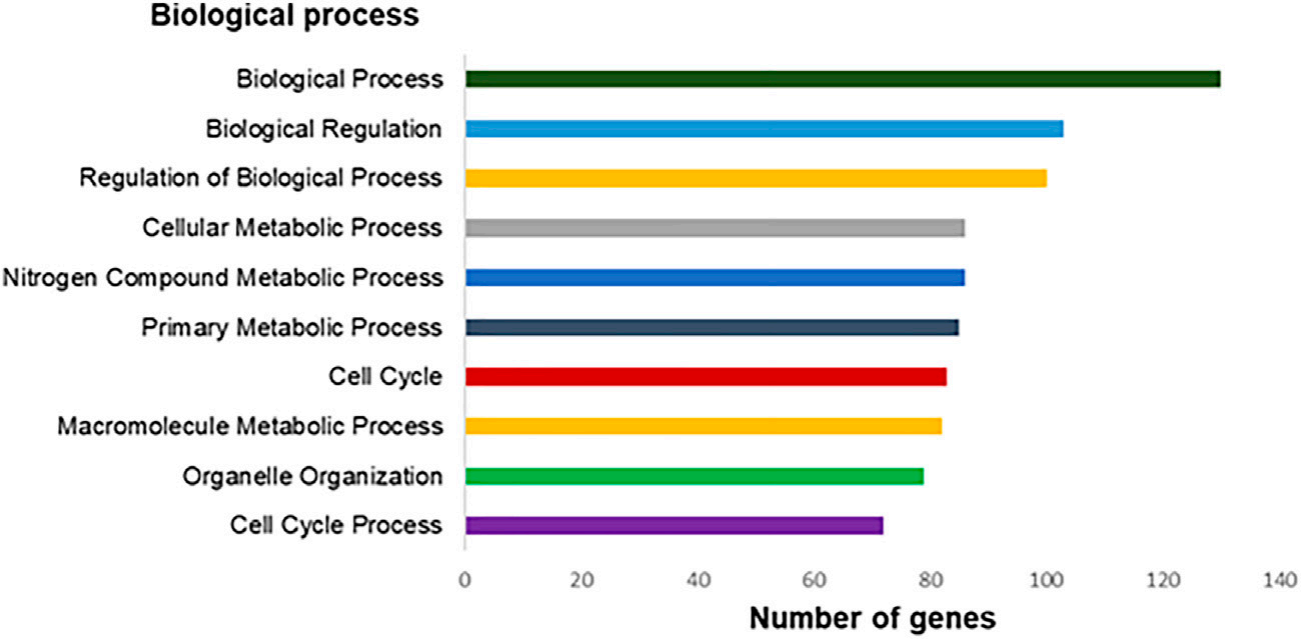
Top-ranking of biological processes based on Gene Ontology annotation (*p* < 0.05) related to differentially expressed genes in the exposed human salivary gland cells line to 2.5 µM of MeHg vs. nonexposed cells (MeHg vs. control).

The genes with expression significantly altered are involved in 672 different biological processes (*p* < 0.05). Among them, we highlighted 10 processes, as *Biological Process, Biological Regulation, Regulation of Biological Processes, Nitrogen Compound Metabolic Process, Cellular Metabolic Process*, and others five processes as shown in (Figure 5).

## Discussion

The human salivary gland cell line exposed to the nonlethal concentration of 2.5 µM MeHg revealed upregulated genes associated with pathways related to genomic stability such as DNA repair and replication, cell-cycle progression and cellular defenses (Table 2), while all downregulated genes were linked to the metabolic process. All these gene profile changes fully explain the metabolic and oxidative impairment in these cells exposed to MeHg (Nogueira et al., 2021), and suggest mechanisms related to the integrity of DNA through activation of DNA repair, integrity, and replication pathways.

**TABLE 2:** Enrichment of pathways and ontologies in upregulated or downregulated in the exposed group of human salivary gland cells line to MeHg.

Pathway	**N**	**NE**	**P.DE**
KEGG Pathways_dowregulated genes (MeHg vs. control)			
Glycosphingolipid biosynthesis-ganglio series	15	1	0.012

KEGG Pathways_upregulated genes (MeHg vs. Control)			
Cell cycle	124	16	3.77^−18^
Progesterone-mediated oocyte maturation	99	8	8.34^−08^
Oocyte meiosis	128	8	6.14^−07^
DNA replication	36	5	1.80^−06^
Cellular senescence	156	7	2.94^−05^
p53 signaling pathway	73	4	0.0008
Nucleotide excision repair	47	3	0.002
FoxO signaling pathway	131	4	0.006
Mismatch repair	23	2	0.007
Human T-cell leukemia virus 1 infection	219	5	0.008
Platinum drug resistance	73	3	0.008
Homologous recombination	41	2	0.023
Caffeine metabolism	5	1	0.028
Viral carcinogenesis	204	4	0.030
Fanconi anemia pathway	54	2	0.039
Pyrimidine metabolism	57	2	0.043
Glutathione metabolism	57	2	0.043

According to the enrichment of gene pathways and ontologies, all the 15 downregulated genes in human salivary gland cells exposed to MeHg are related to glycosphingolipid (GSLs) biosynthesis—ganglio series pathway. The GSLs are amphipathic molecules consisting of a ceramide lipid moiety linked to a glycan chain, in which gangliosides participate in many metabolic processes including growth, differentiation, migration, and apoptosis through modulating both cell signaling processes and cell-to-cell and cell-to-matrix interactions (Lopez and Schnaar, 2009), besides the immune system, cancer progression, and nervous system (Daniotti and Iglesias-Bartolomé, 2011). Alterations in energy availability trigger cell-cycle checkpoints, suggesting a bidirectional connection between cell division and general metabolism. In the exposed cells, cellular senescence pathway had a high number of altered genes. This pathway is important to limit the replication the replication in damaged cells (Van Nguyen et al., 2007; Herranz and Gil, 2018).

The second most downregulated gene was DEPP1, an autophagy regulator. The autophagy process reduces oxidative damage through self-degenerative mechanisms to remove misfolded proteins and damaged organelle, such as mitochondria (Salcher et al., 2017). Thus, the downregulation of DEPP1 may have collaborated to the oxidative imbalance detected in human salivary gland cells line after 2.5 µM MeHg exposure, occurring through the depletion of GSH:GSSG, and increasing of lipid peroxidation and protein carbonyl levels but no damage on DNA (Nogueira et al., 2021).

Although the human salivary gland cell lines had a significant decrease of GSH:GSSG ration after 2.5 µM MeHg exposure, there is an evidence of maintenance of basal glutathione. This is observed through the upregulated genes in the enrichment of gene pathways glutathione (GSH), the major thiol antioxidant, represents the key molecular targets involved in MeHg toxicity, responsible for detoxification, and/or metabolism of a significant number of potentially harmful molecules (Dringen et al., 2015). A recognized gene for maintaining GSH basal levels was upregulated in the exposed cells: ChaC glutathione specific gamma-glutamylcyclotransferase 2 (ChaC2). ChaC family proteins consist of two different branches represented by ChaC1 and ChaC2. While ChaC1 evolves high catalytic efficiency for carrying out an acute glutathione turnover under stress conditions (Kaur et al., 2017), the ChaC2 proteins are responsible for the continuous, but the basal, slow turnover of cytosolic glutathione, representing an additional pathway in the glutathione degradation (Kaur et al., 2017).

The absence of DNA alterations in exposed cells was elucidated by the transcriptome analysis performed here, revealing upregulated genes associated with pathways directly responsible for maintaining the DNA repair, integrity, and replication. The pathways were nucleotide excision repair, mismatch repair, homologous recombination, and DNA replication. Nucleotide excision repair removes bulky DNA lesions coordinated with other aspects of cell metabolism (Schärer, 2013), while the mismatch repair is a highly conserved biological pathway that plays a crucial role in maintaining genomic stability (Li, 2008). The homologous recombination repairs DNA double-stranded breaks (DSBs) and interstrand crosslinks (ICLs) damages, providing massive support for DNA replication in the recovery of stalled or broken replication forks, contributing to the tolerance of DNA damage (Li and Heyer, 2008). One of the important genes identified as upregulated in the exposed cells was the polymerase theta (POLQ). This gene is a widely conserved DNA polymerase and mediates a double-strand break (DSB) repair pathway (Feng et al., 2019).

Based on the KEGG pathway, the higher number of altered genes belongs to the cell cycle pathway. The cell cycle comprises a reproducible sequence of events, DNA replication (S phase), and mitosis (M phase) separated temporally by gaps in the G1 and G2 phases. The most overexpressed gene related to cell cycle arrest in the exposed cells was the cell division cycle 45 (CDC45). The CDC45 gene plays a critical role in DNA replication, assembling the replicative helicase Cdc45-MCM2-7-GINS (CMG), which forms in early S-phase (Speck, 2016). The arresting cell cycle progression responds to the cellular perception of extrinsic factors, such as oxidative stress (Van Nguyen et al., 2007; Herranz and Gil, 2018; Zhao et al., 2020), and is only released followed by complete DNA repair. Otherwise, cells with nonrepairable DNA damage undergo apoptosis (Martin et al., 2010). However, in the exposed human salivary gland cell line, it is possible to observe the upregulation of a gene associated to the apoptosis suppressor, the DNA damage-induced apoptosis suppressor (C11orf82). This gene also named DNA damage-induced apoptosis suppressor (DDIAS), negatively regulates any process of intrinsic apoptotic signaling pathways that shows in response to DNA damage.

Studies suggest that MeHg-induced cell cycle arrest occurs via both p53-dependent and p53-independent pathway manners, but cell death is highly dependent on p53 (Gribble et al., 2005). In exposed stem cells, lethal concentrations of MeHg (20–40 µM) plus an elevated nonlethal concentration (15 µM) actively regulated the p53 pathways (Waldmann et al., 2017). In this present study, although the p53 pathway was active in the exposed human salivary gland cell line, no change in the p53 gene expression profile was observed. However, although Waldman and coauthors (2017) associated p53 to apoptotic response, the p53 pathway is related to DNA repair, maintaining the dynamic equilibrium between cell growth and arrest in response to factors including DNA damage (Mandinova and Lee, 2011), mechanisms that directly agree with the results from exposed salivary gland cell line to MeHg.

The FoxO signaling pathway was also demonstrated after the enrichment of gene pathways and ontologies analysis. This pathway belongs to a family of transcription factors that are activated by oxidative stress signals and regulate cell proliferation and resistance to oxidative stress (Webb and Brunet, 2014). Among the overexpressed genes in the exposed salivary gland cell line, we can point out the forkhead box M. This gene belongs to FoxO signaling pathway and is responsible for protecting cells from the adverse effects of oxidative stress by upregulating the expression of scavenger enzymes. Besides that, this gene participates in the homologous recombination repair of DNA double-strand breaks, preventing polyploidy and aneuploidy (Wierstra, 2013).

Last, one of the main goals after analyzing the overall genes in a biological model is to identify possible candidates of cytotoxicity biomarkers that will better interpret transcriptomics data. A study published by Waldmann and coauthors (2017) identified genes in the stem cells related to lethal concentrations after two different compounds exposure, the MeHg and valproic acid. Comparing to our data, only one similar gene was found altered in the human salivary cells line after exposure to the MeHg, the gene Serpin family F Member 1 (SERPIN1). However, differently from Waldmann’s study, the SERPIN1 gene was downregulated in the present study. This absence of cytotoxic biomarker similarities was already expected since the MeHg concentration used here was nonlethal for cells, validating the Waldmann and coauthors cytotoxicity biomarkers list (2017).

Taking together, the results from the transcriptome profile and our previous study revealed physiological and genomic alterations caused by 2.5 µM MeHg in the human salivary gland cell line. Although nonlethal, this concentration was able to generate oxidative stress and induce different strategies to repair and maintain viable cells. The stress generated by the extrinsic factor (MeHg) triggers changes in gene pathways, directly and indirectly, related to DNA integrity and biochemical responses, such as GSH metabolism. Last, the absence of a common signature of gene expression changes for lethal concentrations of MeHg supports profound studies on gene identifications in nonlethal concentrations xenobiotics.

## Data Availability

The authors acknowledge that the data presented in this study must be deposited and made publicly available in an acceptable repository, prior to publication. The data presented in the study are deposited in the Gene Expression Omninus repository, accession number GSE182249.
